# Hospitals in the United States

**Published:** 1905-11-04

**Authors:** 


### HOSPITAL ADMINISTRATION.

### CONSTRUCTION AND ECONOMICS.

### HOSPITALS IN THE UNITED STATES.

### II. THE MASSACHUSETTS GENERAL HOSPITAL.

By our Special Commissioner.

The Massachusetts General Hospital is far and away the oldest institution of its kind in the commonwealth. It is much to the credit of all who are connected with this great hospital that its general efficiency should be maintained for over a hundred years at the high average which an inspection shows to be everywhere present. It is open, however, to the general criticism that those who have been responsible for the construction and extension of the various buildings do not appear to have been alive to the absolute importance of adequate superficial and wall space in adition to cubic space. In several of the wards there are too many beds, and the tendency to erect verandahs and diminish free superficial space on that portion of the site where the famous old wooden pavilions are still working with complete success, is one which, in our view, ought to be checked or discontinued at the present time. The free circulation of air and an abundance of sunlight are, after all, essential to the successful treatment of acute cases and their rapid recovery.

#### A Touching Memorial to Merit.

The spirit which characterises the management of this hospital secures the confidence of the trained observer from the outset. Twenty-five years ago Dr. Whittemore was medical superintendent. He was amongst the ablest and most popular of hospital superintendents in his day, and he had the sympathy of hospital workers all the world over, owing to the tragedy which ultimately resulted in his death.

Dr. Whittemore instituted many important changes of a beneficial character, and it was very gratifying to notice that everywhere throughout the hospital the notices which intimated these changes bore their original dates still with the signature of Dr. Whittemore. This reprinting and retention of the notices in their original form is deliberate to record for all time that to Dr. Whittemore belonged the credit, and with him the present management wish the credit to remain, always.

We were glad to notice, also, because it is such a satire upon the ruinous extravagance which characterises much of the hospital building in the present day, that the old and famous wooden pavilions are still the most popular parts of the hospital with the surgeons, although they had been put up as a temporary expedient, and were never intended to remain in permanent occupation.

#### Ether Day.

Ether Day, October 16, 1846, is memorable in the history of the institution as being the occasion when the first public demonstration of anæsthesia, to the extent of producing insensibility to pain during a serious surgical operation by the use of sulphuric ether, was demonstrated in the operation theatre. The trustees and managers selected this day as a fitting anniversary of so great an event, far-reaching as it has proved in its benefits to humanity, to throw open for inspection the new out-patient building and other recent additions to the hospital. In addition to the new out-patient department, the cost of which has been mainly defrayed by the generosity of a friend, further donors have presented a ward for the treatment of several classes of cases for which no adequate provision had hitherto been made.

A new power house and domestic building were also erected, and the laundry was reconstructed throughout. In designing these buildings it was determined to so equip them as to make it possible to supply heat, light, and power, not only to the whole institution, but to the Massachusetts Charitable Eye and Ear Infirmary in addition.

It is one of the boasts of the hospital that the out-patient medical and surgical service is, in fact, restricted to such sick and disabled persons as are unable to pay fees, and that no others are ever treated within its walls. A regulation to this effect was enacted on April 1, 1881, being made necessary in consequence of the large and increasing number of persons applying, who were not in need of charitable treatment, and whose attendance obstructed the service of those persons who did need it. In consequence of this regulation it has been apparent to the people of Boston that the Massachusetts General Hospital is a charity intended for the poor only, and all who have attempted to abuse the hospital have been refused service by the physicians and surgeons; and so this Boston hospital makes claim to have solved successfully the question of abuse, which so largely interferes with the efficiency of many of our hospitals in the present time.

#### New Out-patient Department.

There can be no doubt to anyone familiar with hospital construction, who visits the new out-patient department, that very great care and thought have been expended upon the arrangement and general plan. The building consists of three floors and a basement, and is L-shaped in form. The large waiting-room is situated in the right-angle of the initial letter, though the immediate corner is occupied by the staircase and a lift. Facing the visitor on leaving the lift is an out-patient waiting-room having a corridor to the left, which communicates with a large lecture-hall, and on either side of this corridor are placed dressing and examination rooms, etherising and operation rooms, with a recovery room, and at the farther end on the right a large room for dressing septic cases, and on the left one for dressing clean cases. In the longer corridor to the left are placed the records room, known as the desk-room, where probably the most complete system of recording cases, and following the treatment—the maladies of every applicant for admission, no matter how many times he or she may have received relief on previous occasions, are readily produced for the practitioner's information—is carried out with an efficiency and a smartness under the direction of Mrs. Myers, which cannot fail to win the admiration of everybody who spends time in mastering the details. On the left, beyond the desk-room, are lavatories and examination rooms, and the same arrangement prevails to the right of the corridor, with the addition of two class-rooms at the extreme end.

The first floor is devoted to surgical cases, the second to medical cases, and the third to special departments. The equipment of the throat, ear, skin, and electrical departments is so complete as to make them quite remarkable. The cost has, of course, been great. The authorities are convinced that everything they have provided is essential to the efficiency of the work; and as public opinion and private money have supported them in this view, it may be accepted as the opinion locally, whatever the actual opinion of the visitor may, in fact, be. The operation building is very complete. It was gratifying to note that the Bigelow amphitheatre is still very popular, and that it contains the bust of the great surgeon and also his operation chair. Many of the fittings and appliances in the operation building, especially, were of the highest order, and the smartness and general cleanliness and brightness of the whole institution testify to the thoroughness of the supervision, and the efficiency of every member of the staff. The trustees have evidently done their best to restrain the architect from indulging in too much elaboration and costly additions to the exterior of the building; they have not, however, succeeded in preventing him from indulging his tendency to luxury in the very handsome marble-coped exterior staircase at the rear of the out-patients' building.

### Cheap Ward Construction Justified.

It was very interesting to visit once again after an interval of very many years the Bigelow Townsend wards, and to note that the operation theatre in the Bigelow ward was still, on the whole, more popular with the staff than the costly modern operation buildings. Certainly these old wards have done splendid service, and are to-day in many respects as attractive and suitable for the treatment of patients as any wards which may be mentioned. They are certainly, in our judgment, even more efficient than a number of wards in costly buildings which have been erected within recent years.

### A Complete System of Manufacture.

A system has grown up at the Massachusetts General Hospital under Dr. Howard, the present able medical superintendent, which is in many respects unique. He has organised a workshop, and set up machinery which supplies to the patients, and even to other institutions, and to outside members of the medical profession for their patients, all kinds of surgical appliances, including artificial limbs. The workmanship is excellent, the type of many of these artificial aids to locomotion is novel and better than can be met with elsewhere, and the cost of them is about one-third of what it would be if they were obtained in the ordinary way. Dr. Howard, with the aid of Dr. Washburn, his assistant, has set up some machinery for washing and purifying aseptic dressings, and reconverting them for use for hospital purposes. The departments in the basement are all under the supervision of one trained engineer, and each has at its head a skilled mechanic. All the electro-plating, and, indeed, every kind of repair, and most of the new furniture and appliances, such as dressing-trays, operation tables, trolleys, and an infinity of other appliances, are made on the premises. In the result a large saving in the annual expense is effected.

### A Swedish Gymnasium.

There is also a complete and very extensive Swedish gymnasium, full of apparatus for every purpose, where four trained assistants are kept actively employed each day, to the no small comfort and benefit not only of the patients who frequent the hospital, but of many people of a better class who need the treatment and pay for it. The latter would not be received as hospital patients. Certainly the old Massachusetts General Hospital exhibits a vitality and an output of energy and enthusiasm which entitles it to the grateful recognition of the people of Boston, and will, we have no doubt, secure for it all the funds which its trustees and managers may need for its efficient maintenance and upkeep.

